# Structural and Biochemical Characterization Reveals LysGH15 as an Unprecedented “EF-Hand-Like” Calcium-Binding Phage Lysin

**DOI:** 10.1371/journal.ppat.1004109

**Published:** 2014-05-15

**Authors:** Jingmin Gu, Yingang Feng, Xin Feng, Changjiang Sun, Liancheng Lei, Wei Ding, Fengfeng Niu, Lianying Jiao, Mei Yang, Yue Li, Xiaohe Liu, Jun Song, Ziyin Cui, Dong Han, Chongtao Du, Yongjun Yang, Songying Ouyang, Zhi-Jie Liu, Wenyu Han

**Affiliations:** 1 Key Laboratory of Zoonosis, Ministry of Education, College of Veterinary Medicine, Jilin University, Changchun, China; 2 Shandong Provincial Key Laboratory of Energy Genetics, Qingdao Institute of Bioenergy and Bioprocess Technology, Chinese Academy of Sciences, Qingdao, China; 3 Center for Biological Imaging, Institute of Biophysics, Chinese Academy of Sciences, Beijing, China; 4 National Laboratory of Biomacromolecules, Institute of Biophysics, Chinese Academy of Sciences, Beijing, China; National Jewish Medical and Research Center, United States of America

## Abstract

The lysin LysGH15, which is derived from the staphylococcal phage GH15, demonstrates a wide lytic spectrum and strong lytic activity against methicillin-resistant *Staphylococcus aureus* (MRSA). Here, we find that the lytic activity of the full-length LysGH15 and its CHAP domain is dependent on calcium ions. To elucidate the molecular mechanism, the structures of three individual domains of LysGH15 were determined. Unexpectedly, the crystal structure of the LysGH15 CHAP domain reveals an “EF-hand-like” calcium-binding site near the Cys-His-Glu-Asn quartet active site groove. To date, the calcium-binding site in the LysGH15 CHAP domain is unique among homologous proteins, and it represents the first reported calcium-binding site in the CHAP family. More importantly, the calcium ion plays an important role as a switch that modulates the CHAP domain between the active and inactive states. Structure-guided mutagenesis of the amidase-2 domain reveals that both the zinc ion and E282 are required in catalysis and enable us to propose a catalytic mechanism. Nuclear magnetic resonance (NMR) spectroscopy and titration-guided mutagenesis identify residues (e.g., N404, Y406, G407, and T408) in the SH3b domain that are involved in the interactions with the substrate. To the best of our knowledge, our results constitute the first structural information on the biochemical features of a staphylococcal phage lysin and represent a pivotal step forward in understanding this type of lysin.

## Author Summary

The staphylococcal phage lysin LysGH15 demonstrates great potential against methicillin-resistant *Staphylococcus aureus* (MRSA). Here, we report that the lytic activity of LysGH15 and its CHAP domain is dependent on calcium ions. To elucidate the molecular mechanism, we determined the structures of three individual LysGH15 domains using X-ray crystallography or nuclear magnetic resonance (NMR). The crystal structure unexpectedly reveals an “EF-hand-like” calcium-binding site near the Cys-His-Glu-Asn quartet active site groove in the LysGH15 CHAP domain. Furthermore, the calcium ion plays an important role as a switch that modulates the lytic activity of the CHAP domain. Additionally, structure-guided mutagenesis also confirms that both E282 and the zinc ion play an important role in maintaining the lytic activity of the LysGH15 amidase-2 domain. Moreover, the NMR structure and titration-guided mutagenesis identify residues in the LysGH15 SH3b domain that are involved in the interactions with the substrate. The structure of LysGH15 is the first determined lysin structure from a staphylococcal phage, and these results represent a pivotal step forward in understanding this type of lysin.

## Introduction

Although *Staphylococcus aureus* is a common habitant of the human skin and respiratory tract, several highly pathogenic strains are major causes of hospital-associated infections and can be life threatening, particularly in immunocompromised patients [1]. Over the past three decades, the incidence of methicillin-resistant *S. aureus* (MRSA) infection, particularly as caused by community-associated MRSA (CA-MRSA) isolates, has dramatically increased worldwide, which raises serious concerns within the medical community [2]. USA300 is the most prevalent CA-MRSA strain and accounts for up to 97% of all CA-MRSA infections [3]. The treatment of infections caused by CA-MRSA has become increasingly difficult due to the emergence of multidrug resistance [4]. Therefore, an urgent need exists for novel therapeutic agents directed against this formidable pathogen [5], [6].

Lysin (also known as endolysin) is a cell wall hydrolase that is synthesized at the end of the phage lytic life cycle and is involved in cell lysis and the release of progeny particles from host cells [7]. Lysin can also rapidly and specifically lyse Gram-positive bacteria when exogenously applied [8]. Because the bacterial cell wall is conserved and is necessary for the life cycle, the current lack of reports on the development of bacterial resistance against lysin is not surprising [9]. Additionally, the species- or type-specificity guarantees that the lysin will not affect the normal microflora [10]. Thus, lysin is thought to be a promising potential antibacterial agent.

In our previous study, we reported that LysGH15, which is encoded by the staphylococcal phage GH15, demonstrates strong lytic activity against MRSA *in vitro* and *in vivo* [11], [12]. LysGH15 shares very high sequence identity with other lysins of class III staphylococcal phages, such as LysK, phi11, and MV-L [13]. Moreover, these lysins possess a modular structure containing an N-terminal CHAP domain (cysteine, histidine-dependent amidohydrolases/peptidases), a central amidase-2 domain (N-acetylmuramoyl-L-alanine amidase), and a C-terminal SH3b domain (the SH3 bacterial binding domain, which typically contains 60–70 residues and is homologous to eukaryotic SH3 proteins) [14]–[17]. CHAP domains as well as amidase-2 domains are types of catalytic domains commonly found in lysins. Although the potent lytic activity against MRSA has been extensively characterized, the molecular mechanism of LysGH15 and of other homologous staphylococcal phage lysins has remained unclear.

In this study, we report that the lytic activity of the LysGH15 CHAP domain is critically dependent on calcium ions. To elucidate the molecular mechanism, we determined the structures of three individual LysGH15 domains. The crystal structure of the CHAP domain unexpectedly reveals a calcium-binding site near the Cys-His-Glu-Asn quartet active site (the active site that consists of cysteine, histidine, glutamic acid, and asparagine typically found in members of the CHAP family [18]), and site-directed mutagenesis further confirms that both the calcium ion binding site and the conserved Cys-His-Glu-Asn quartet are required for the lytic activity of the LysGH15 CHAP domain. Structure-based mutagenesis also confirms that E282 and the zinc ion play an important role in maintaining the lytic activity of the amidase-2 domain. Furthermore, NMR titration-guided mutagenesis identifies the potential target binding interface of the LysGH15 SH3b domain. These details provide a pivotal step forward in understanding this type of lysin.

## Results

### The activity of each individual catalytic domain

**Figure 1:**
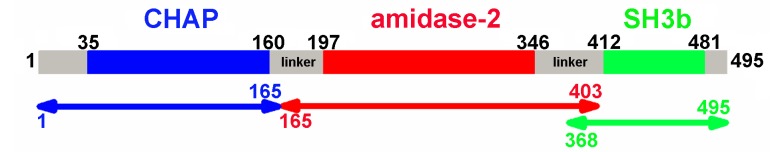
Domain organization of LysGH15. LysGH15 contains three domains: the CHAP domain (blue, residues 35–160), the amidase-2 domain (red, residues 197–346), and the SH3b domain (green, residues 412–481). Two linkers (residues 161–196 and 347–411) are located between the three domains. The lines with two arrows indicate the regions that were crystallized or analyzed using NMR.

Bioinformatic analysis (PSIPRED [19]) and known domain boundaries led us to define the CHAP construct as residues 35–160, the amidase-2 construct as residues 197–346, and the SH3b construct as residues 412–481 in LysGH15 (**Figure 1**). According to this information, we designed several constructs of each individual domain. Some constructs could be expressed and purified as soluble proteins in *Escherichia coli*. Each individual domain eluted as a single peak during size-exclusion chromatography (SEC) (**Figure S1A**). Sedimentation velocity experiments using analytical ultracentrifugation (AUC) indicated that the three individual domains all exist as monomers in solution (**Figure S1B**).

The activity of each individual catalytic domain was determined (**Table 1**). The wild type (wt) CHAP domain alone also demonstrates bactericidal activity, but this activity is much weaker than that of full-length LysGH15, and a high concentration (50 µM) is required to achieve a similar bactericidal effect as the full-length LysGH15 (0.25 µM). At concentrations below 1 µM, nearly no bactericidal activity for the CHAP domain is detected. Moreover, the lytic activity of the isolated CHAP domain requires a longer time, in contrast to the extremely rapid lysis of the CA-MRSA strain USA300 by full-length LysGH15. The amidase-2 domain of LysGH15 does not demonstrate lytic activity. Surprisingly, the amidase-2 domain is able to enhance the lytic activity of the CHAP domain against USA300 (**Table 1**).

**Table 1:** The bactericidal activity of the different constructs.

	The velocity of the decrease in OD_600_ (OD/min)
Control	5.9×10^−4^
CHAP (10 µM)	28.0×10^−4^
CHAP (50 µM)	53.6×10^−4^
amidase-2 (50 µM)	5.1×10^−4^
CHAP (50 µM)+amidase-2 (50 µM)	64.4×10^−4^
CHAP (50 µM)+E282A-amidase-2 (50 µM)	52.8×10^−4^
CHAP (0.25 µM)+SH3b (0.25 µM)	5.7×10^−4^
LysGH15 (0.25 µM)	69.0×10^−4^
C54S-LysGH15 (0.25 µM)	5.9×10^−4^
C54S-LysGH15 (0.25 µM)+CHAP (0.25 µM)	7.6×10^−4^
E282A-LysGH15 (0.25 µM)	66.4×10^−4^
D45A-LysGH15 (0.25 µM)	5.5×10^−4^
D47A-LysGH15 (0.25 µM)	5.4×10^−4^
Y49A-LysGH15 (0.25 µM)	65.0×10^−4^
H51A-LysGH15 (0.25 µM)	67.0×10^−4^
D56A-LysGH15 (0.25 µM)	5.7×10^−4^
H214A-LysGH15 (0.25 µM)	63.0×10^−4^
H324A-LysGH15 (0.25 µM)	63.5×10^−4^
C332A-LysGH15 (0.25 µM)	65.0×10^−4^
N404A-LysGH15 (0.25 µM)	21.7×10^−4^
Y406A-LysGH15 (0.25 µM)	30.5×10^−4^
(N404A+Y406A)-LysGH15 (0.25 µM)	23.3×10^−4^
G407A-LysGH15 (0.25 µM)	35.5×10^−4^
T408A-LysGH15 (0.25 µM)	12.8×10^−4^
(G407A+T408 A)-LysGH15 (0.25 µM)	11.3×10^−4^
S430A-LysGH15 (0.25 µM)	62.3×10^−4^
L433A-LysGH15 (0.25 µM)	49.3×10^−4^
(S430A+L433A)-LysGH15 (0.25 µM)	44.6×10^−4^
I454A-LysGH15 (0.25 µM)	42.4×10^−4^

The initial OD_600_ value of the bacteria was approximately 1.0. Different proteins were added into the bacteria solution, and the OD_600_ was kinetically measured for 120 min.

**Figure 2:**
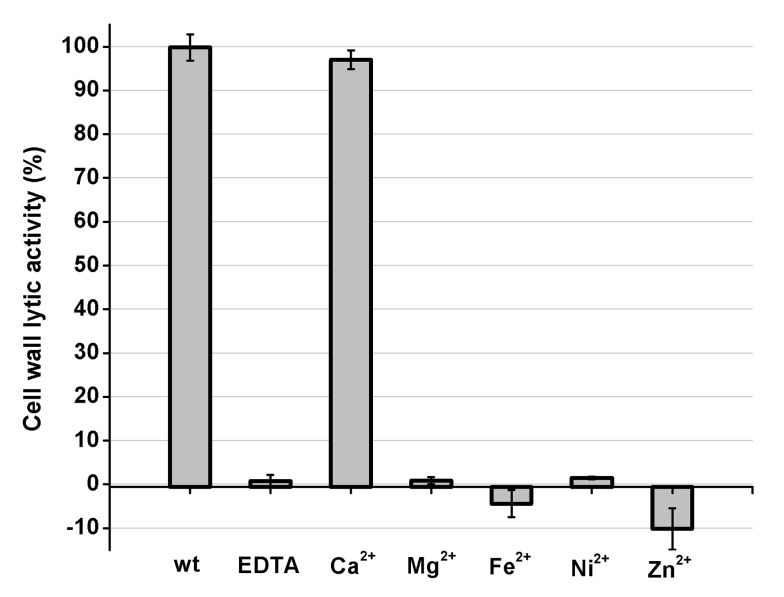
Cell wall lytic activity of the LysGH15 CHAP domain. The effects of different ions (5 µM) on the lytic activity of the EDTA-inactivated CHAP (5 µM) domain (excess EDTA was removed by dialysis). The values represent the mean ± SD (n = 3).

PSI-BLAST analysis using the National Center for Biotechnology Information (NCBI) database reveals that the amidase-2 domain contains a conserved zinc-binding site. To determine the role of the zinc ion in the activity of the amidase-2 domain, the combination of the CHAP and amidase-2 domains was pretreated with EDTA (1 mM), and excess EDTA was removed by dialysis. Surprisingly, the lytic activity of the combination was completely abolished. Additionally, the individual CHAP domain is also sensitive to EDTA (**Figure 2**), which suggests that the lytic activity of the CHAP domain is dependent on the presence of metal ions. However, the PSI-BLAST analysis does not indicate a conserved ion-binding site in the CHAP domain or in homologous proteins. Therefore, we sought to determine the structure of LysGH15, particularly that of the CHAP domain, and further elucidate the nature of this phenomenon.

### Structure determination

Full-length LysGH15 was expressed in *E. coli*, but the protein exhibited degradation. Although the introduction of a C-terminal His-tag improved the stability of LysGH15, the slow degradation of LysGH15 was not amenable to crystallization, which hindered our initial attempts to crystallize full-length LysGH15. However, the crystallization of individual LysGH15 domains was possible, and the respective structures were successfully determined (**Table S1**). The X-ray crystal structures of the CHAP and amidase-2 domains were determined using the selenium single-wavelength anomalous dispersion (Se-SAD) and iodide single-wavelength anomalous dispersion (I-SAD) methods, respectively, due to the lack of homologous structures. Crystals of the individual CHAP domain encompassing residues 1–165 and of the amidase-2 domain encompassing residues 165–403 were obtained, as shown in **Figure 1**. Additionally, the three-dimensional structure of the SH3b domain was determined using NMR spectroscopy in solution (**Table S2**). The NMR spectra of the SH3b domain were acquired using a construct containing residues 368–495 (**Figure 1**).

### The CHAP domain contains a calcium-binding site

**Figure 3:**
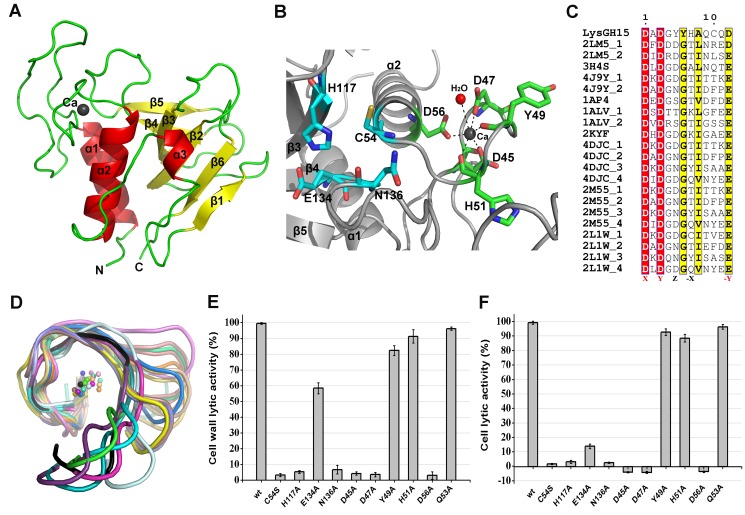
Structural and functional analysis of the LysGH15 CHAP domain. (**A**) Overall structure of the CHAP domain (residues 1–164). β-strands and α-helices are numbered. The N- and C-termini are labeled with the respective letters. The Ca^2+^ ion in the LysGH15 CHAP domain is shown as a black sphere. (**B**) A detailed view of the catalytic site (cyan) and the calcium-binding site (green) in the CHAP domain. (**C**) Sequence alignment of the 12-residue calcium-binding site. The first four characters indicate the PDB code except for the LysGH15 CHAP domain. The positions 1, 3, 5, 7, and 12 are indicated by X, Y, Z, -X, and –Y, respectively. The alignment was generated using CLUSTAL W (http://www.ch.embnet.org/software/ClustalW.html). The figure was generated using ESPript (http://espript.ibcp.fr/ESPript/ESPript/index.php). (**D**) Structural comparison of the 12-residue calcium-binding sites shown in (**C**). The calcium ions are shown as spheres. The calcium-binding site of the LysGH15 CHAP domain is shown in black. The cell wall catalytic (**E**) and bactericidal (**F**) activities of the CHAP domain containing different mutations. The concentrations of the proteins used in this study are 50 µM (live USA300 cells) and 5 µM (cell wall), respectively. The values represent the mean ± SD (n = 3).

The CHAP domain crystallizes in the *P*6_2_22 space group with two molecules in the asymmetric unit (ASU). Iterative rounds of model building followed by refinement result in a model with good statistics and geometry (**Table S1**). The quality of the electron density permits the unambiguous modeling of residues 1–164. The final model is refined to 2.69 Å resolution (*R*_work_ = 17.37%, *R*_free_ = 20.39%). The two molecules form a dimer that is mediated by hydrogen bonding interactions at the central interface by one molecule of Bis-Tris-propane that was present in the crystallization reservoir solution. However, a Bis-Tris-propane molecule does not mediate homodimer formation of the CHAP domain in solution and cannot affect the activity of the CHAP domain. The CHAP domain forms a globular structure that is comprised of three α-helices that are packed against six β-sheets, as shown in **Figure 3A**.

Notably, as shown in **Figure 3B**, residues D45, D47, Y49, H51, and D56 coordinate a central Ca^2+^ ion that forms the classical 12-residue (positions 1, 3, 5, 7, and 12) calcium-binding site. The presence of the calcium was further confirmed using inductively coupled plasma atomic emission spectrometry (ICP-AES) analysis. The side chains of D45, D47, and D56 and the main chains of Y49 and H51 are 2.36, 2.37, 2.13, 2.32, and 2.27 Å, respectively, from the Ca^2+^ ion, as shown in **Figure 3B** and **Table 2**. Additionally, the coordination sphere of the calcium-binding site is completed by a water molecule (**Figure 3B**). The isothermal titration calorimetry (ITC) demonstrated that the equilibrium dissociation constant of the LysGH15 CHAP domain for Ca^2+^ is approximately 27 µM (**Figure S2**). The circular dichroism (CD) spectroscopy showed that the presence/absence of Ca^2+^ does not affect the secondary structures of the LysGH15 CHAP domain (**Figure S3A**). However, the fluorescence-based thermal shift assays showed ∼2°C shift in *Tm* of the CHAP domain with/without Ca^2+^, which indicated that Ca^2+^ has a slight contribution to the protein thermostability (**Figure S3B**).

**Table 2:** Ca^2+^-oxygen ligands in the LysGH15 CHAP domain.

	Residue	Atom	Distance (Å)	Key residues for Ca^2+^ binding^a^
X (1)	D45	OD1	2.36	•
Y (3)	D47	OD1	2.37	•
Z (5)	Y49	O	2.13	○
-X (7)	H51	O	2.32	○
-Y (12)	D56	OD2	2.27	•
Average (Å)			2.29	
(Second molecule)			(2.27)	
B-factor of Ca^2+^ (Å)			47.41	
(Second molecule)			(48.51)	

a •: indicates the key residue for calcium binding in the LysGH15 CHAP domain; ○: indicates nonessential residues for calcium binding in the LysGH15 CHAP domain.

**Figure 4:**
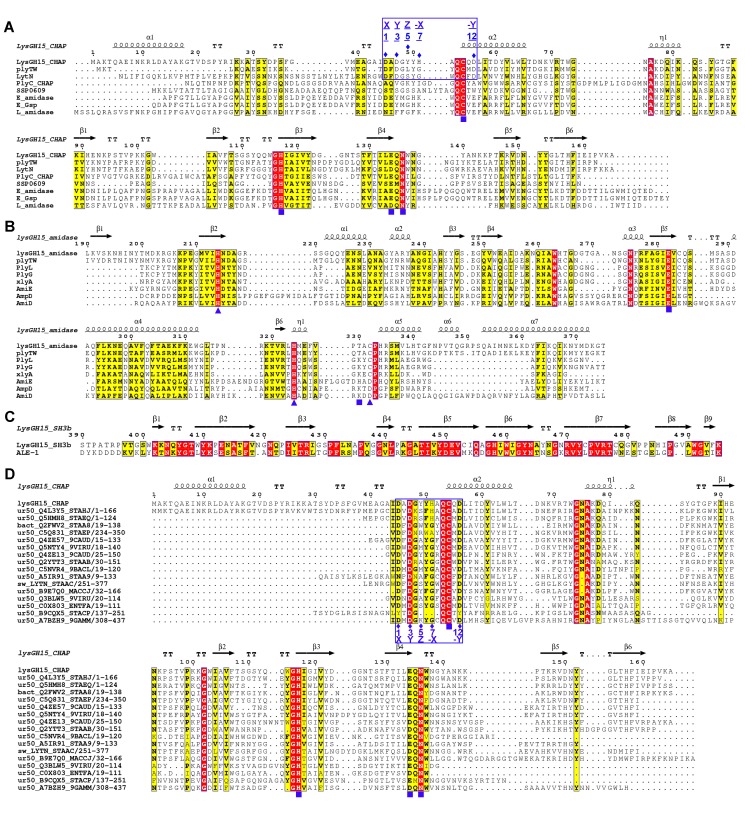
Sequence alignment of the three individual LysGH15 domains and homologous proteins. (**A**) The CHAP domain. The 12-residue calcium-binding site is indicated by a blue box. The Cys-His-Glu-Asn quartet and the calcium-binding residues (conserved positions 1, 3 5, 7, and 12) are indicated by filled blue squares and filled blue diamonds, respectively. (**B**) The amidase-2 domain. The zinc-binding residues of this domain are indicated by filled blue triangles, and the catalytic residues are indicated by filled blue squares. (**C**) The SH3b domain. (**D**) Sequence alignment of the CHAP domain with several members of the CHAP family from the Pfam database. The accession number of this family in the Pfam database is PF05257 (http://pfam.sanger.ac.uk/family/PF05257). The Cys-His-Glu-Asn quartet residues are indicated by filled blue squares. The 12-residue calcium-binding site is indicated by a blue box, and the positions 1, 3, 5, 7, and 12 (filled blue diamonds) are indicated by X, Y, Z, –X, and –Y, respectively. All alignments were generated using CLUSTAL W (http://www.ch.embnet.org/software/ClustalW.html). The figure was generated using ESPript (http://espript.ibcp.fr/ESPript/ESPript/index.php). Strictly conserved residues are boxed in white on a red background, and highly conserved residues are boxed in black on a yellow background. A schematic representation of the secondary structure elements of the corresponding structures are shown above the sequences.

**Figure 5:**
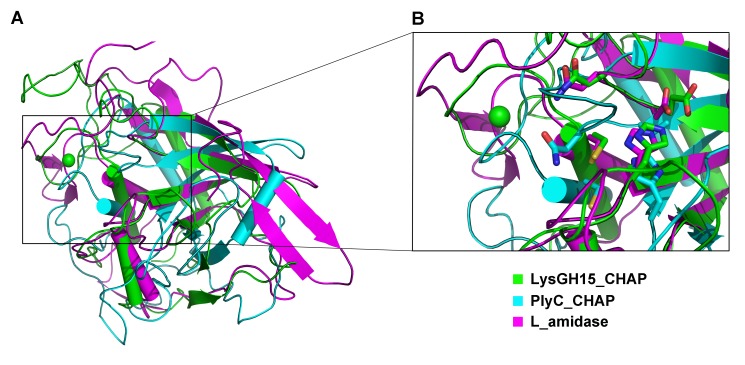
Structural comparison of the LysGH15 CHAP domain with homologous proteins. (**A**) Representation of the overall fold. Helices are shown as cylinders, and strands are shown as arrows. The calcium ion in the active site of the LysGH15 CHAP domain is shown as a green sphere. PlyC_CHAP, PDB ID: 4F88 [21]; L_amidase, PDB ID: 2VPS [65]. (**B**) A detailed view of the superposed Cys-His-Glu-Asn quartet sites and the calcium-binding site of the LysGH15 CHAP domain.

Sequence-based searches reveal that the CHAP domain shares very little identity (<28%) (**Figure 4A**) with the proteins that are deposited in the Protein Data Bank (PDB); therefore, the CHAP family represents a rare example of a protein family that is defined by a unique family member. In contrast, searches for structurally similar proteins in the PDB using the DALI server [20] produces several hits of sizeable Z-scores, as shown in **Table 3**. The most structurally homologous protein is the CHAP domain of PlyC [21], which is a streptococcal-specific phage lysin (Z-score = 11.4, Root-mean-square deviation (RMSD) = 2.32 Å). Additionally, the LysGH15 CHAP domain also possesses a fold that is similar to the structures of *Staphylococcus saprophyticus* SSP0609 and several other proteins (**Figure 5A**, **Table 3**). The superposition of the LysGH15 CHAP domain with these proteins indicates the putative peptidoglycan-binding groove as demonstrating the highest similarity, particularly the Cys-His-Glu-Asn quartet (**Figure 5B**). Surprisingly, none of these structurally homologous proteins contains a calcium-binding site corresponding to the position of the calcium-binding site in the LysGH15 CHAP domain (**Figure 5B**), which indicates that the calcium-binding site of the LysGH15 CHAP domain is unique. Therefore, the CHAP domain of LysGH15 represents a sub-family of the CHAP family.

**Table 3:** Structural comparison of each individual domain with structurally homologous proteins using the DaliLite server.

Three domains of LysGH15	Homologous proteins	DALI score	RMS deviation	Number of aligned Cα atoms	Total residues (range)	PDB ID
CHAP	*Streptococcus* phage C1 endolysin PlyC (PlyC_CHAP)	11.4	2.32	121	155 (310–464)	4F88
	*Staphylococcus saprophyticus* CHAP domain (SSP0609)	6.2	2.83	93	115 (41–155)	2K3A
	*Staphylococcus aureus* staphyloxanthin biosynthesis protein	7.7	3.04	95	113 (1–113)	2LRJ
	*Escherichia coli* glutathionylspermidine synthetase/amidase (E_amidase)	8.4	2.66	110	193 (10–202)	2IO8
	*Escherichia coli* K-12 glutathionylspermidine synthetase/amidase (E_Gsp)	8.2	2.71	110	190 (8–197)	3A2Z
	*Leishmania* trypanothione synthetase-amidase (L_amidase)	8.4	2.61	101	182 (2–183)	2VPS
amidase-2	*Bacillus anthracis* prophage endolysin PlyL	21.2	1.62	146	157 (1–157)	1YB0
	*Bacillus* phage gamma amidase PlyG	19.5	1.76	146	165 (165)	2L47
	*Bacillus subtilis* amidase xlyA	19.4	1.63	133	154 (1–154)	3HMB
	*Staphylococcus epidermidis* autolysin AmiE	17.7	2.24	154	207 (7–213)	3LAT
	*Citrobacter freundii* amidase AmpD	12.7	2.42	132	179 (1–179)	2Y2B
	*Escherichia coli K-12* amidase AmiD	8.5	3.93	155	257 (5–261)	2WKX
SH3b	*Staphylococcus capitis* EPK1 peptidylglycan hydrolase ALE-1	13.1	1.56	91	98 (264–362)	1R77

The sequence and structure of the calcium-binding site in the LysGH15 CHAP domain were compared with the 12-residue calcium-binding sites from randomly selected proteins in the PDB. As shown in **Figure 3C**, residues at positions 1 and 3 are highly conserved and are frequently aspartate, and the residue at position 12 is generally glutamate (an aspartate in the LysGH15 CHAP domain), whereas positions 5 and 7 demonstrate greater deviation among these proteins. From the structures shown in **Figure 3D**, the initial half of the “annulus” loops is similar among the different proteins, whereas the terminal half of the loops exhibit large deviations.

### The calcium ion modulates the activity of the CHAP domain

To identify the metal ion that critically affects the lytic activity of the CHAP domain, common metal ions were added to the EDTA-inactivated CHAP domain. Interestingly, we found that the loss of the lytic activity can be restored only when calcium is added, whereas the addition of other ions, such as magnesium, iron, or zinc, is not able to activate the lytic activity of this domain (**Figure 2**). This finding indicates that the lytic activity of the CHAP domain is specifically dependent on calcium. An identical behavior was also detected for LysGH15, which led to the question of whether the calcium ion that critically affects the lytic activity is coordinated by these five residues.

To answer this question, residues D45, D47, Y49, H51, and D56 were individually mutated to alanine. The lysis assay indicated that the D45A, D47A, and D56A mutations all result in a significant loss of bactericidal and cell wall catalytic activity, as shown in **Figure 3E** and **Figure 3F**. Moreover, a unique calcium spectrometry signal is not detected in the D45A, D47A, or D56A mutant proteins using ICP-AES (**Table 2**). Furthermore, supplementation with calcium does not restore the activity of these three mutants. In contrast, the activities of the Y49A and H51A mutants marginally decrease (i.e., these mutants retain >80% of the activity). Additionally, the Q53A mutation was constructed as a control that retains lytic activity. Collectively, these results indicate that the calcium ion bound by these five residues is necessary for the lytic activity of the CHAP domain, and residues D45, D47, and D56 play an important role in coordinating this calcium ion.

Additionally, analysis of the molecular conservation of the CHAP domain using ConSurf [22] indicates several highly conserved residues at or near the surface, such as D47, Q53, C54, D56, G74, N75, H117, E134, and N136, which form a narrow and deep groove (**Figure S4A**). The architecture and size of this groove indicate that it most likely serves as the binding site for a portion of the peptidoglycan. In the center of the groove, C54, H117, E134, and N136 form the Cys-His-Glu-Asn quartet. C54 is positioned at the beginning of helix α1, followed by H117 at the beginning of strand β3, E134 at the end of strand β4, and N136 proximal to the beginning of the loop that links strands β4 and β5 (**Figure 3B**). To assess the roles of these four residues, mutated LysGH15 CHAP domains were expressed and purified. We found that the C54A, C54S, H117A, and N136A mutations in the CHAP domain result in a loss of bactericidal and cell wall hydrolytic activity. The mutant E134A only retains a portion of the lytic activity (E134A retains ∼58% activity in the cell wall and ∼15% in cells), as shown in **Figure 3E** and **Figure 3F**. These results indicate that this quartet plays an important role in the lytic activity of the CHAP domain. Coincidentally, the calcium ion that is present within this active site lies particularly close to C54. However, ICP-AES analysis indicates that the C54A and C54S mutations do not affect the calcium binding of the CHAP domain.

Moreover, a C54S mutation in the full-length LysGH15 results in the complete loss of lytic activity, as shown in **Table 1**. Therefore, the CHAP domain primarily determines the lytic activity of LysGH15. However, the C54S LysGH15 mutant (0.25 µM) does not demonstrate lytic activity even with complementation using the native CHAP domain (0.25 µM). It is likely that the high lytic activity of LysGH15 cannot be ascribed to the CHAP domain alone. Additionally, D45A, D47A, or D56A mutations in LysGH15 also result in the complete loss of lytic activity (**Table 1**), which further indicates that the lytic activity of LysGH15 is dependent on the calcium.

### Residues that are essential for the activity of the amidase-2 domain

**Figure 6:**
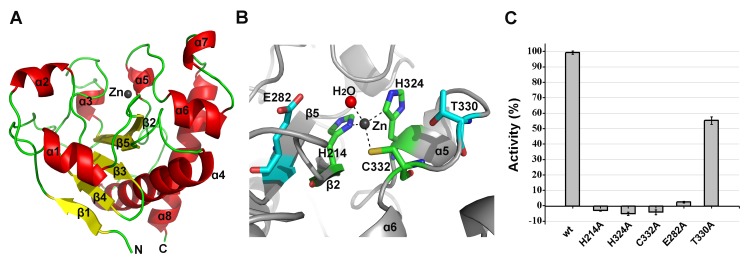
Structural and functional analysis of the LysGH15 amidase-2 domain. (**A**) The overall structure of the amidase-2 domain (residues 189–373). β-strands and α-helices are numbered. The N- and C-termini are labeled with the respective letters. The Zn^2+^ ion in the LysGH15 amidase-2 domain is shown as a black sphere. (**B**) A detailed view of the catalytic site (cyan) and the zinc-binding site (green) in the amidase-2 domain. Zinc is shown as a black sphere. (**C**) The ability of the amidase-2 domain (50 µM) containing different mutations to enhance the lytic activity of the CHAP domain (50 µM). The values represent the mean ± SD (n = 3).

The structure of the amidase-2 domain (residues 165–403) was determined at 2.27 Å resolution. As shown in **Figure 6A**, the amidase-2 domain exhibits a ββααββαβααααα topology. Electron density for 24 residues at the N-terminus and 30 residues at the C-terminus is absent. A recessed area located on the surface of this structure is enclosed by helices α2, α3, α5, α7 and several loops. β2, β3 and β5 form the bottom of this recessed area. One zinc ion is located at the center of this groove and interacts with the side chains of residues H214, H324, and C332, as shown in **Figure 6B**. The tetrahedral coordination sphere of zinc is completed by a water molecule, which is an arrangement often observed in the active sites of zinc-dependent metalloenzymes. The architecture and size of the groove indicate that it most likely serves as the binding site for a portion of the peptidoglycan (**Figure S4B**). This potential active site is solvent exposed and lies within a shallow groove on the protein surface, which is consistent with its ability to cleave a highly crosslinked and branched polymer.

**Figure 7:**
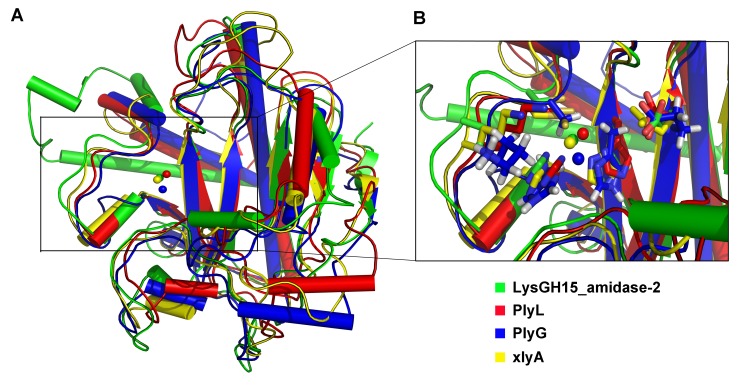
Structural comparison of the LysGH15 amidase-2 domain with homologous proteins. (**A**) Representation of the overall fold. Helices are shown as cylinders, and strands are shown as arrows. Zinc ions in the active sites are shown as spheres. PlyL, PDB ID: 1YB0 [23]; PlyG, PDB ID: 2L47 (Dias, J.S. et al., unpublished results); and xlyA, PDB ID: 3HMB [66]. (**B**) A detailed view of the active sites and zinc-binding sites. Zinc ions are shown as spheres.

We compared the structure of the LysGH15 amidase-2 domain with other proteins available in the PDB, as shown in **Figure 7A**. Despite similar folds, the amino acid sequences of these proteins share very little identity (<25%) with the LysGH15 amidase-2 domain. Indeed, the closest protein is the amidase domain of PlyL from *Bacillus* phage (PDB ID: 1YB0) [23], which shares only approximately 20% sequence identity (**Figure 4B**) and an RMSD value of 1.62 Å for the LysGH15 amidase-2 domain (**Table 3**). Several proteins that exhibit structural similarity with the LysGH15 amidase-2 domain are summarized in **Table 3**. All of these structures share the α1–α5 helices and all of the β-strands (β1–β5). In particular, β2, β5, α5, and L_α5α6_ are well conserved because they contain the zinc-binding sites and conserved residues, including N275 and E282.

Mutation of the three zinc-binding residues to alanine results in a complete loss of the activity (i.e., the ability to enhance the lytic activity of the CHAP domain) of the LysGH15 amidase-2 domain, as shown in **Figure 6C**. E282 in the LysGH15 amidase-2 domain lies in an identical position as E90 in PlyL and E93 in xlyA (**Figure 7B**), which places it in a subclass of the *N*-acetylmuramoyl-L-alanine amidases [23]. Site-directed mutagenesis indicates that the activity of the E282A mutant is completely lost (**Figure 6C**). These results indicate that both the zinc-binding site and E282 are necessary for the activity of the LysGH15 amidase-2 domain. However, the E282A mutation or mutation of the zinc-binding residues (H214A, H324A, or C332A) does not affect the lytic activity of full-length LysGH15 (**Table 1**). T330 in LysGH15 is structurally homologous to K135 in PlyL and K128 in the T7 lysozyme (histidine in AmiE). The finding that the T330A mutant demonstrates a 50% decrease in enhancing ability for the CHAP domain suggests that T330 is also involved in the activity of the amidase-2 domain. N275 in the LysGH15 amidase-2 domain is an additional conserved residue, which corresponds to N112 in the autolysin AmiE.

### The NMR structure of the SH3b domain and the identification of residues involved in binding

**Figure 8:**
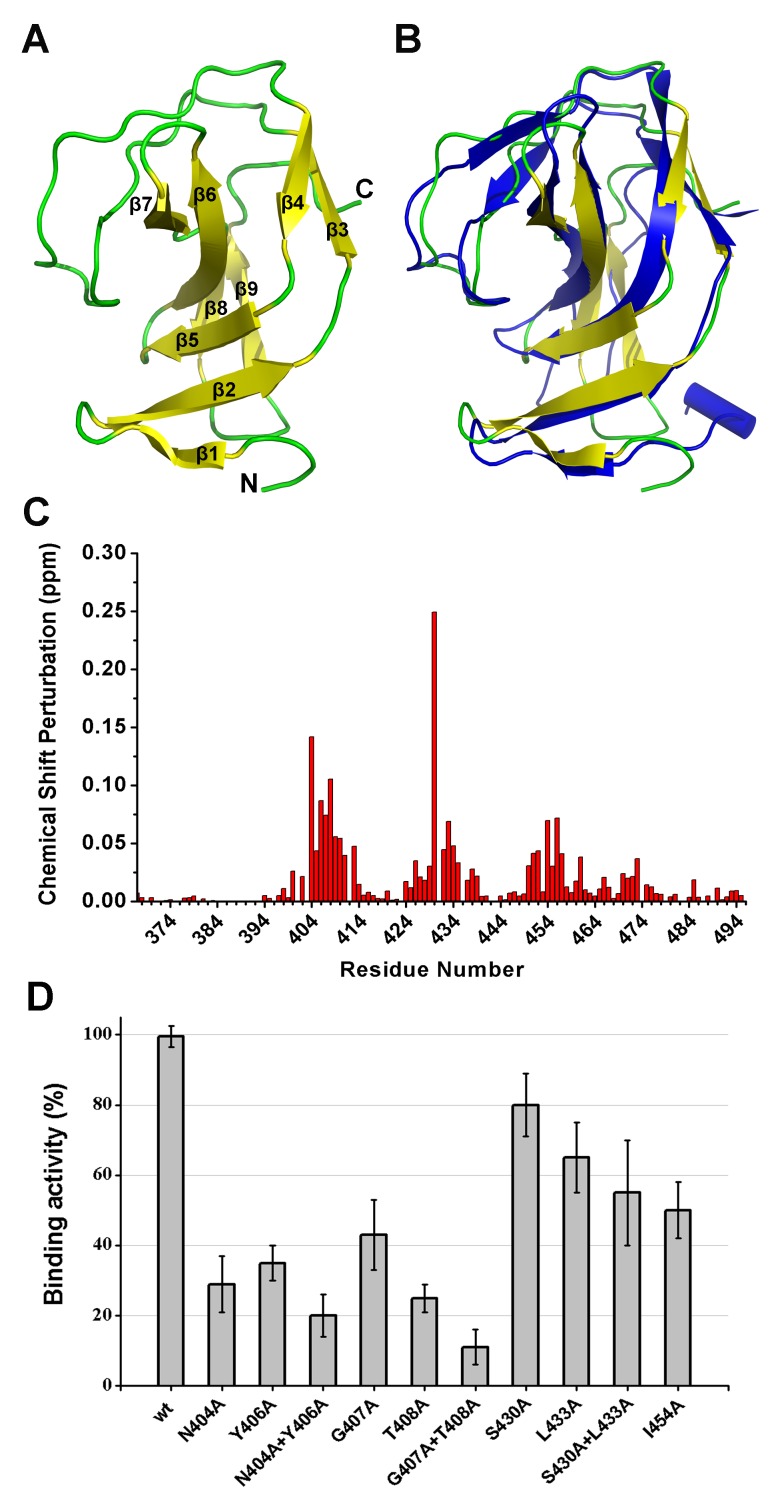
Structural and functional analysis of the LysGH15 SH3b domain. (**A**) Overall structure of the SH3b domain (residues 400–495). The β-strands are numbered. The N- and C-termini are labeled with the respective letters. (**B**) Structural comparison of the LysGH15 SH3b domain (yellow) with the binding domain (blue) of ALE-1 (PDB ID: 1R77 [24]). (**C**) Chemical shift perturbations in the peptide (“AGGGGG”) titration versus the residue number of the SH3b domain. (**D**) Binding of the SH3b domain and its mutants to *S. aureus* USA300 peptidoglycan. Bound protein (mutant) to peptidoglycan was detected using ELISA and normalized against the wt SH3b domain.

Although the construct of the SH3b domain contains residues 368–495, the determined NMR structure indicates that residues 400–495 form a compact domain, whereas residues 368–399 form a flexible linker. The SH3b domain consists of nine β-strands from residues 400–495, as shown in **Figure 8A**. All β-strands are antiparallel. Several loops between the β-strands (i.e., L_β1β2_, L_β2β3_, L_β6β7_, and L_β7β8_) exhibit larger RMSD values compared with the β-strand regions in the structure, suggesting that these loops exhibit higher flexibility. The overall structure of the LysGH15 SH3b domain is very similar to the SH3b domain of the ALE-1 protein (which is a peptidoglycan hydrolase produced by *Staphylococcus capitis* EPK1 that can specifically lyse *S. aureus*) (**Figure 8B**) [24]. These proteins share approximately 47% sequence identity (**Figure 4C**).

A comparison of the structures of the two SH3b domains reveals a major difference in that the residues of ALE-1 corresponding to β7 and β8 of the LysGH15 SH3b domain adopt a loop conformation. The loop L_β7β8_ and strands β7 and β8 pack against β2 and β5 in the LysGH15 SH3b domain, whereas the corresponding region in the ALE-1 SH3b domain exists as a loop that points toward the outside of the protein. In the sequence alignment, two additional proline residues are present in the L_β7β8_ loop of the LysGH15 SH3b domain, and these proline residues participate in hydrophobic interactions with residues in β2 and β5, which may explain this structural difference. Additionally, the construct used in the determination of the ALE-1 SH3b domain structure contained an N-terminal FLAG-tag, which occupies the region corresponding to the L_β7β8_ loop of the LysGH15 SH3b domain.

In light of the sequence and structural similarities with the SH3b domain of ALE-1, in addition to an identical target bacteria (*S. aureus*), the peptide “AGGGGG” was used to perform NMR titrations of the ^15^N-labeled LysGH15 SH3b domain, as previously reported for the SH3b of ALE-1 [24]. Significant chemical shift perturbations (CSPs) in a fast-exchange manner (on the NMR timescale) upon peptide addition are observed in the ^1^H-^15^N HSQC spectra of the LysGH15 SH3b domain, as shown in **Figure 8C** and **Figure S5A**. The equilibrium dissociation constant *K*_D_ is approximately 3.0 mM, as obtained by fitting the titration curve of the CSPs (**Figure S5B**), which indicates that the binding is quite weak and may explain the inability to cocrystallize the ALE-1 SH3b domain with the polyglycine peptide in a previous study [24].

Residues exhibiting large CSP values are clearly clustered in the sequence, and mapping of the CSP results onto the structure of the LysGH15 SH3b domain reveal that these residues may interact with the peptide. The residues with significant CSPs are largely from β1, β2, β5, loop L_β1β2_, and L_β3β4_, which form a groove for peptide binding (**Figure S4C** and **Figure S5C**). The potential binding site is consistent with the polyglycine binding site of the ALE-1 SH3b domain that was proposed by previous docking and mutagenesis analysis [24], [25]. However, S430 is the residue with the most significant CSP in the amidase domain of LysGH15 (the corresponding residue in ALE-1 is G299).

To determine the effect of these residues on the binding, these residues were mutated to alanine in the individual SH3b domain of LysGH15. As shown in **Figure 8D**, the mutations N404A, Y406A, G407A, or T408A in the SH3b domain significantly diminish the binding activity. Additionally, the mutations L433A and I454A also affect the binding of the SH3b domain. Although S430 exhibits a large CSP value in the NMR titrations, S430A does not substantially affect the activity of the SH3b domain, which indicates that the side chain of S430 may not be involved in the binding. The large chemical shift perturbation of S430 may be caused by the main chain of G429 that participates in the binding: the carbonyl of G429 points into the binding groove and may form a hydrogen bond with the HN of the peptide, which induces a large chemical shift of the S430 HN. Additionally, the effects of these residues on the lytic activity of the full-length LysGH15 were investigated. From **Table 1**, the bactericidal activity of LysGH15 is reduced when these residues (except with S430) are mutated to alanine.

## Discussion

In this study, we report that the lytic activity of the LysGH15 and its CHAP domain is critically dependent on a calcium ion. To confirm these findings, the structures of the individual CHAP, amidase-2, and SH3b domains of LysGH15 were determined. This study represents the first report of the structure of a staphylococcal phage lysin.

Interestingly, the structural studies reveal that the CHAP domain of LysGH15 contains a calcium-binding site that is located near the active site groove. This finding is unexpected because the calcium-binding site is not detectable through sequence-based searches alone [26]. To the best of our knowledge, LysGH15 is the first characterized lysin that contains a calcium-binding site. However, LysGH15 is not the first lysin to demonstrate calcium-dependent lytic activity. Although we have investigated the calcium-dependence of LysGH15 in our previous study [11], the LysGH15 used was not pretreated with EDTA, which masked the detection of this important phenomenon. It has been reported that the LysK CHAP domain [27], the staphylococcal phi11 lysin [28], the streptococcal B30 lysin [29], and the streptococcal Ply700 lysin [30] exhibit calcium-dependence.

A classical EF-hand protein contains a helix–loop–helix Ca^2+^-binding motif. The “EF-hand-like” motif differs from the classical EF-hand as follows: (i) the length of the Ca^2+^-binding loop is shorter or longer than 12 residues and/or (ii) the secondary structure elements of the flanking regions are not two helices [26]. Although the calcium-binding loop of the LysGH15 CHAP domain is 12 residues in length (the coordination residues lie at positions 1, 3, 5, 7, and 12), the secondary structure elements surrounding the calcium-binding site of the CHAP domain only contain one helix, which is consistent with an “EF-hand-like” motif. Thus, the LysGH15 CHAP domain represents an “EF-hand-like” protein. As in the protective antigen from *Bacillus anthracis* (PDB ID: 1ACC) [31] and the dockerin from *Clostridium thermocellum* (PDB ID: 1DAQ) [32], the calcium-binding site of the LysGH15 CHAP domain lacks the exiting helix, forming a “loop-F” pattern. Notably, the functions of these proteins available in the PDB that contain a calcium-binding site are completely unrelated to those of the LysGH15 CHAP domain. To date, the CHAP domain of LysGH15 is the first identified “EF-hand-like” protein that originates from a phage lysin.

As in the thermolysin-like protease, the calcium ion plays an important role as a switch that modulates the protease between active and inactive states according to the biological demand [33]. Note that D45, D47, and D56 coordinate the calcium ion via their side chains, whereas Y49 and H51 coordinate the calcium ion via their main chains. Therefore, it is not surprising that the activity of the LysGH15 CHAP domain is retained upon mutation of Y49A and H51A. The reason for the calcium-dependence of LysGH15 is not clear. However, in light of its location near the active site groove and its significant influence on the lytic activity of the protein, there are two potential functions for this calcium ion: (i) it participates in the catalytic activity as part of the reaction; or (ii) it positions the key residues, particularly C54, to form the appropriate conformation. The C54S/C54A mutation completely abolishes the lytic activity, which indicates that the sulfhydryl of C54 acts as a nucleophile and plays a critical role in the hydrolysis. This finding is consistent with the conclusion obtained from studies on the *E. coli* glutathionylspermidine (GSP) synthetase, which operates via a nucleophilic mechanism involving C59 as the catalytic nucleophile [34].

Some members (6.56%, 366/5579) in the Pfam sequence database (http://pfam.sanger.ac.uk/family/PF05257), which exhibit high sequence identity with the LysGH15 CHAP domain in the calcium-binding site (particularly at positions 1, 3, and 12) and in the Cys-His-Glu-Asp proteolytic active site (several sequences are provided in **Figure 4D**), are likely to also coordinate calcium, and their hydrolytic activity is expected to be dependent on calcium. Additionally, 36.04% (71/197) of the members in the family C51 of the MEROPS peptidase database (http://merops.sanger.ac.uk/index.shtml) also exhibit this sequence identity.

As was observed for the inactive staphylococcal Φ11 lysin [15], the streptococcal λSA2 lysin *N*-acetylglucosaminidase domain [35], [36], and LysK [37], [38], the LysGH15 amidase-2 domain alone is silent during activity analysis. Nevertheless, the LysGH15 amidase-2 domain is not entirely silent but additionally exhibits the ability to enhance the lytic activity of the CHAP domain. Therefore, the amidase-2 domain may cleave specialized substrates of the peptidoglycan, such as the bond between *N*-acetylmuramoyl-L-alanine [37]. The two domains may be able to simultaneously cleave the peptidoglycan between D-alanine and glycine, as well as Mur-NAc and L-alanine [37]. These large defects in the superstructure of the cell wall would result in rapid bacterial lysis [21].

The complex of AmiD with its substrate has been described, and the catalytic mechanism of AmiD has been elucidated [39]. Although the sequences of the LysGH15 amidase-2 domain and AmiD are divergent, they exhibit homologous structures and share conserved active site residues. In AmiD, E104 (E282 in the LysGH15 amidase-2) and a zinc ion activate the water that is bound to the zinc ion, which favors the nucleophilic attack of the amide bond; the tetrahedral intermediate is stabilized by K159 (this site is occupied by T330 in the LysGH15 amidase-2 domain). Mutational analysis demonstrates that E282 and T330 are critical for the activity of the LysGH15 amidase-2 domain. Thus, the amidase-2 domain of LysGH15 most likely possesses a similar catalytic mechanism as AmiD. Asparagine has been reported to be the only residue conserved among bacterial and eukaryotic amidases that participates in peptidoglycan binding [1]. Here, we find that the amidase-2 domain of LysGH15 from phage (virus) also contains this conserved residue (i.e., N275) at a corresponding position as other amidase members. These results provide further evidence for the hypothesis that “a common peptidoglycan binding mode is shared by all proteins with an *N*-acetylmuramyl-L-alanine amidase-like fold” [1].

Although the SH3b domain does not possess lytic activity [14], in light of the large difference in the activity of full-length LysGH15 and its CHAP domain alone, the SH3b domain is expected to be necessary for LysGH15 to display high processive activity. The NMR titration indicates that the residues exhibiting large CSP values formed a deep and narrow groove. Additionally, most of these residues significantly affect the binding activity of the SH3b domain and the lytic activity of LysGH15. We assume that the binding of the SH3b domain to its cognate receptor is dominant in localizing the catalytic domain to the cell wall. Once the catalytic domain is positioned close to the peptidoglycan layer, the local concentration of substrate is greatly enhanced, and significant catalysis may ensue [40]. Thus, both the binding activity of the SH3b domain and the catalytic activity of the CHAP contribute to the high processivity of LysGH15.

## Materials and Methods

### Constructs, protein expression, and purification

The genes for the full-length LysGH15 and its three individual domains (i.e., CHAP, amidase-2, and SH3b) were amplified using corresponding primers that were designed based on the full-length *lysGH15* gene (GenBank: AY176327) and were synthesized by Sangon Biotech (Shanghai) Co., Ltd. The coding regions for the CHAP (residues 1–165), amidase-2 (residues 165–403), and SH3b (residues 368–495) domains were cloned into the pMCSG7 vector as previously reported [41]. The full-length *lysGH15* gene was subcloned into the pET-26b vector. Mutations were designed based on these constructs and were generated using the QuikChange Site-Directed Mutagenesis Kit following the manufacturer’s instructions (Stratagene). All of the recombinant plasmids were sequenced to verify the sequence.

The plasmids harboring the target gene, which encoded 6× His-tagged proteins, were transformed into *E. coli* BL21(DE3) (Tiangen Biotechnology). The cells were grown in Luria-Bertani (LB) medium at 37°C until the OD_600_ reached 0.8. The culture was then induced with 0.2 mM isopropyl-β-D-thiogalactoside (IPTG) for 20 h at 16°C. Cells were harvested by centrifugation at 4,670×g for 30 min and were resuspended in phosphate-buffered saline (PBS; 137 mM NaCl, 2.7 mM KCl, 50 mM Na_2_HPO_4_, and 10 mM KH_2_PO_4_, pH 7.4). After lysis by sonication, the cell debris was removed by centrifugation at 38,900×g for 30 min. The supernatant was applied to a nickel-nitrilotriacetic acid (Ni-NTA) resin gravity column (Qiagen) that had been previously equilibrated with PBS. The column was washed using 100 ml of lysis buffer containing 20 mM imidazole, followed by a 50 mM imidazole wash. Finally, the protein was eluted with PBS containing 500 mM imidazole. After buffer exchange, the 6× His-tag was removed using tobacco etch virus (TEV) proteolysis (except for full-length LysGH15). Uncut protein was removed using a second Ni-affinity chromatography step. The proteins without a His-tag were concentrated and applied to a Superdex G200 size-exclusion chromatography column (Amersham) that was preequilibrated with 20 mM Tris-HCl (pH 7.5) and 150 mM NaCl (500 mM NaCl for full-length LysGH15). For the SH3b domain, 40 mM Na_3_PO_4_ and 50 mM NaCl, pH 6.5, were used. Fractions containing the purified target protein were pooled and stored at −80°C until further analysis.

The *E. coli* BL21(DE3) strain that contained the pMCSG7-CHAP vector was grown in M9 medium containing glucose (0.2% M/V), MgSO_4_ (1 mM), and ampicillin (100 µg/ml) at 37°C until the OD_600_ reached 0.8. Subsequently, selenomethionine was added to the culture (50 µg/ml). The subsequent purification steps were similar to those used for the native protein.

The plasmid pMCSG7-SH3b was transformed into *E. coli* BL21(DE3). The cells were grown in M9 medium containing glucose (0.2% M/V), MgSO_4_ (1 mM), and ampicillin (100 µg/ml). ^15^N ammonium chloride and/or ^13^C glucose was used as the sole nitrogen and carbon sources, respectively, for isotope labeling. Labeled SH3b was purified using an identical procedure as that used for the native protein.

### Crystallization, NMR, data collection and processing, and structure determination

All of the proteins were initially screened for crystallization using the hanging-drop vapor diffusion method and commercially available sparse matrix screens at 16°C. Crystals were obtained by mixing 1 µl of the protein solution with an equal volume of the reservoir solution and equilibrating the mixed drop against 300 µl of the reservoir solution. Crystals of CHAP and Se-Met-CHAP were grown in a solution containing 0.1 M Bis-Tris-propane, pH 7.5, and 3.8 M sodium formate using 10–15 mg/ml of the proteins. The crystal of the amidase-2 domain was grown using 10–18 mg/ml of the proteins and a reservoir solution containing 0.1 M Tris-HCl, pH 9.0, 0.2 M Li_2_SO_4_, 30% (wt/vol) PEG 3,000, and 0.1 M xylitol. For phase determination, crystals of amidase-2 were soaked for 5 min in crystallization solution supplemented with 10 mM potassium iodide (KI) prior to cryoprotection and freezing.

Diffraction data for native amidase-2 domain crystals and crystals soaked with KI were collected at beamline BL5.0.1 (Advanced Light Source, Lawrence Berkeley National Laboratory, USA). Otherwise, diffraction data for the native CHAP domain crystal and anomalous diffraction data for the selenomethionine CHAP domain crystal were collected at 100 K using an ADSC Q315 CCD detector at beamline BL17U1 of the Shanghai Synchrotron Radiation Facility (SSRF). The data sets were indexed, integrated, and scaled using the HKL2000 software [42]. The initial phases were determined using the X^2^DF structure determination pipeline [43], [44] and the Se-SAD method [45], and the initial model was built by PHENIX AutoBuild [46]. The models were manually improved in Coot [47]. Refinement was alternately performed using REFMAC [48] and PHENIX Refine [46]. Statistics for the data collection and refinement are summarized in **Table S1**.

NMR samples of the SH3b domain (labeled by ^15^N and/or ^13^C) contained 0.02% (w/v) sodium 2,2-dimethylsilapentane-5-sulfonate (DSS) and 10% (v/v) ^2^H_2_O. All NMR experiments were performed at 298 K on an Agilent DD2 600 MHz NMR spectrometer that was equipped with a Z-gradient triple-resonance cryoprobe, as previously described with some modifications [49]. Two-dimensional ^1^H-^15^N and ^1^H-^13^C HSQC and, three-dimensional CBCA(CO)NH, HNCACB, HNCO, HN(CA)CO, HBHA(CO)NH, HCCH-TOCSY, and CCH-TOCSY experiments were performed for SH3b backbone and side chain assignments. Three-dimensional ^1^H-^15^N and ^1^H-^13^C NOESY-HSQC spectra with mixing times of 150 ms were collected to generate distance restraints. All data were processed using NMRPipe [50] and were analyzed using NMRViewJ [51]. Proton chemical shifts were referenced to the internal DSS, and ^15^N and ^13^C chemical shifts were referenced indirectly [52]. The structures of SH3b were initially calculated using the program CYANA [53] and were then refined using CNS [54] with manual assignments as well as semi-automated NOE assignments performed using SANE [55]. Backbone dihedral angle restraints that were obtained using TALOS-N [56] and hydrogen-bond restraints according to the regular secondary structure patterns were also incorporated into the structural refinement. From the 100 initial structures, 50 of the lowest energy conformers of SH3b were selected for water refinement using CNS and RECOORDScript [57], and the 20 lowest energy conformers were selected to represent the final ensemble of structures for SH3b. The quality of the structures was analyzed using MOLMOL [58] and PROCHECK-NMR [59]. Statistics for the data collection and refinement are summarized in **Table S2**.

### Catalytic activity of the full-length LysGH15 and individual domains

The CA-MRSA strain USA300-TCH1516 was obtained from the American Type Culture Collection (ATCC) and was used throughout the study. Staphylococcal lytic assays were performed using an overnight culture of USA300 grown at 37°C in tryptic soy broth (TSB) supplemented with 1% wt/vol yeast extract. Staphylococci were washed in PBS (pH 7.4) and resuspended at an OD_600_ of approximately 1.0. Bacteria were mixed with the wt or mutant proteins, and the OD_600_ was kinetically measured in a spectrophotometer for 120 min. All assays were performed in triplicate. Peptidoglycan was isolated from stationary phase cultures of USA300, as previously described with some modifications [1]. For a quantitative analysis of lysis, purified peptidoglycan from USA300 was dissolved in 50 mM sodium phosphate buffer and was adjusted to an OD_578_ of approximately 0.6. When the protein was processed using EDTA (1 mM), the excess EDTA was removed by dialysis. The peptidoglycan lytic activity was measured as the decrease in the OD_578_ for 60 min.

### Titration of the SH3b domain with the peptide “AGGGGG”

The peptide “AGGGGG” was synthesized by Sangon Biotech (Shanghai) Co., Ltd. A stock solution (100 mM) of the peptide was prepared in a buffer that was identical to that used for the SH3b protein sample. The interaction between SH3b and the peptide was detected by monitoring the two-dimensional ^1^H-^15^N HSQC spectra of SH3b during the titration. The observed CSPs were calculated as previously described [49] using the following formula:
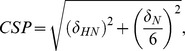
where δ_HN_ and δ_N_ are the changes in the ^1^H_N_ and ^15^N chemical shifts, respectively. The equilibrium dissociation constants (*K*_D_) were estimated by fitting the CSPs to the following equation:
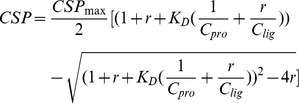
where CSP_max_ is the CSP at the theoretical saturated condition, which was also obtained from the fit; r is the molar ratio of the peptide to the protein; C_pro_ is the concentration of the initial protein solution; and C_lig_ is the stock concentration of the peptide.

### Detection of SH3b binding to bacterial peptidoglycans using ELISA

The protocols of ELISA used here were similar to those previously described with some modifications [24]. Briefly, polystyrene enzyme immunoassay 96-well plates (Nunc PolySorp; Thermo Fisher Scientific) were incubated with 100 µl of sonicated bacterial peptidoglycans at a concentration of 15 µg/ml in PBS at 4°C overnight. After coating, the wells were washed three times with distilled water, and the plate was subsequently blocked with 1% bovine serum albumin in PBS at 4°C overnight. The wells were washed three times with distilled water, and 100 µl of 10 µg/ml protein diluted in PBS was added to the wells and incubated at 4°C for 1 h. After incubation with protein, the wells were washed three times with PBS containing 0.05% Tween 20. Anti-SH3b serum (100 µl) diluted in PBS containing 0.1% bovine serum albumin was added to the wells and incubated at 37°C for 1 h. After three washes with PBS-Tween 20, 100 µl of diluted goat anti-rabbit IgG horseradish peroxidase conjugate was added and incubated for 1 h at 37°C. Unbound conjugate was removed by washing three times with PBS-Tween 20. Subsequently, 100 µl of substrate (3,3',5,5'-tetramethylbenzidine solution) was added, and the reaction was stopped by the addition of 100 µl of 1 M H_2_SO_4_. The optical density was measured at 450 nm in a spectrophotometer.

### Analytical ultracentrifugation

Sedimentation velocity experiments were performed using a Beckman XL-I analytical ultracentrifuge at 20°C as previously described [60]. Briefly, protein samples were diluted with buffer (20 mM Tris-HCl, pH 7.5, and 150 mM NaCl) to 400 µl at an absorbance at 280 nm of approximately 0.75. The samples were loaded into a conventional double-sector quartz cell and mounted in a Beckman four-hole An-60 Ti rotor. The data were collected at 262,000×g (at the cell center) at a wavelength of 280 nm. Interference sedimentation coefficient distributions were calculated from the sedimentation velocity data using SEDFIT (www.analyticalultracentrifugation.com).

### ICP-AES analysis

Inductively coupled plasma atomic emission spectrometry (ICP-AES, Varian, VISTA-MPX) was used for the metallic evaluation of protein samples at Tsinghua University, as previously described with modifications [61]. The conditions for ICP-AES analysis were as follows: RF power, 1.15 kW; plasma gas flow rate (Ar), 15 l/min; nebulizer gas flow rate (Ar), 0.75 l/min; auxiliary gas flow rate (Ar), 1.5 l/min; and viewing height, 12 mm. This analysis was performed using three replicates.

### Isothermal titration calorimetry (ITC)

Measurements were conducted at 16°C using an ITC-200 microcalorimeter (GE Healthcare) as described previously [62]. The samples were buffered with 20 mM Tris buffer pH 7.5 containing 200 mM NaCl. To determine the calcium-binding affinity of the CHAP domain, 600 µM CaCl_2_ was stepwise injected into 50 µM the Ca^2+^-free CHAP protein sample. The data were analyzed with the MicroCal Origin software.

### Circular dichroism (CD) spectroscopy

CD spectra were acquired on a Chirascan CD Spectrometer (Applied Photophysics) according to the previous description [63]. Freshly prepared CHAP protein with or without Ca^2+^ was adjusted to 0.15 mg/ml in 20 mM Tris, pH 7.5, 200 mM NaCl prior to the measurements. Wavelength spectra were recorded at 20°C using a 0.1-cm path length cuvette. Each scan was obtained by recording every 1 nm with a bandwidth of 1 nm between the wavelength ranges of 200–260 nm.

### Thermal shift assay

Thermal shift assays were conducted using 0.2 mg/ml of the CHAP protein with or without Ca^2+^ in a buffer [20 mM Tris (pH 7.5), 200 mM NaCl] supplemented with a 1,000 dilution of SYPRO Orange dye (Invitrogen) as described previously [64]. The fluorescence signals as a function of temperature were recorded using a real-time PCR machine (CFX96; Bio-Rad) in the FRET mode, in which the fluorescence intensity was measured with excitation/emmission 450–490/560–580 nm. The temperature gradient was set in the range of 20–95°C with a ramp of 0.5°C over the course of 15 s. Data were analyzed with the differential scanning fluorimetry analysis tool (Excel-based), and the Boltzmann model was used for plotting melting curves of CHAP proteins to obtain the midpoint of the thermal unfolding value for CHAP proteins using the curve-fitting software XL fit 5 (ID Business Solutions Ltd.).

### Protein structure accession numbers

The atomic coordinates and structure factors have been deposited in the Protein Data Bank (PDB) under accession codes 4OLK for the CHAP domain, 4OLS for the amidase-2 domain, and 2MK5 for the SH3b domain. The BMRB accession ID for the SH3b domain is 19752.

### Statistical analysis

Statistical significance was determined using the unpaired two-tailed Student’s *t*-test at a level of significance of P<0.05.

## Supporting Information

Figure S1 **Purification of LysGH15 and its three individual domains.** (**A**) Size-exclusion chromatography (SEC) of the full-length LysGH15 and its three individual domains (CHAP, amidase-2, and SH3b). SDS-PAGE analysis (inset) of the protein collected from each major peak. (**B**) Analytical ultracentrifugation (AUC) analysis of the three individual LysGH15 domains (CHAP, residues 1–165; amidase-2, residues 165–403; and SH3b, 368–495).
(TIF)

Figure S2 **The equilibrium dissociation constant of the CHAP domain in the presence of calcium ions was determined using ITC.** (**A**) 600 µM CaCl_2_ was stepwise injected into 50 µM the CHAP protein samples. (**B**) 600 µM CaCl_2_ was injected into the buffer as control.
(TIF)

Figure S3 **The biophysical effect of Ca^2+^ binding to the LysGH15 CHAP domain.** (**A**) Circular dichroism (CD) spectroscopy of the CHAP domain with/without Ca^2+^. (**B**) The thermal shift assays of the CHAP domain with/without Ca^2+^.
(TIF)

Figure S4 **Alignment of sequences and surface rendering of the groove.** (**A**) The CHAP domain. (Left), alignment of the 35 unique protein sequences indicating the conserved residues; (right), surface rendering of the groove in the LysGH15 CHAP domain. (**B**) The amidase-2 domain. (Left), alignment of the 44 unique protein sequences; (right), surface rendering of the groove in the LysGH15 amidase-2 domain. (**C**) The SH3b domain. (Left), alignment of the 26 unique protein sequences; (right), surface rendering of the groove in the LysGH15 SH3b domain. This figure was generated using the ConSurf server (http://consurftest.tau.ac.il/) and PyMOL [67].
(TIF)

Figure S5 **Titration of the LysGH15 SH3b domain with the peptide “AGGGGG”.** (**A**) ^1^H-^15^N HSQC spectra of the titration of the SH3b domain with the peptide “AGGGGG”. Residues exhibiting chemical shift perturbations are labeled. (**B**) *K*_D_ values for binding of the SH3b domain to “AGGGGG”. The fit of the four residues exhibiting the largest chemical shift perturbations are shown with the determined *K*_D_ values. (**C**) A detailed view of the residues in the SH3b domain that interact with the “AGGGGG” peptide. The residues involved in the interaction with the peptide are shown as sticks.
(TIF)

Table S1 **Data collection and refinement statistics.**
(DOC)

Table S2 **The experimental restraints and structural statistics for the 20 lowest energy structures of the LysGH15 SH3b domain.**
(DOC)
