# Supplementary figures and images for: Structural and Biochemical Characterization Reveals LysGH15 as an Unprecedented “EF-Hand-Like” Calcium-Binding Phage Lysin
Source: PLoS Pathog. 2014 May 15;10(5):e1004109. doi: 10.1371/journal.ppat.1004109 (PMC4022735; doi:10.1371/journal.ppat.1004109)

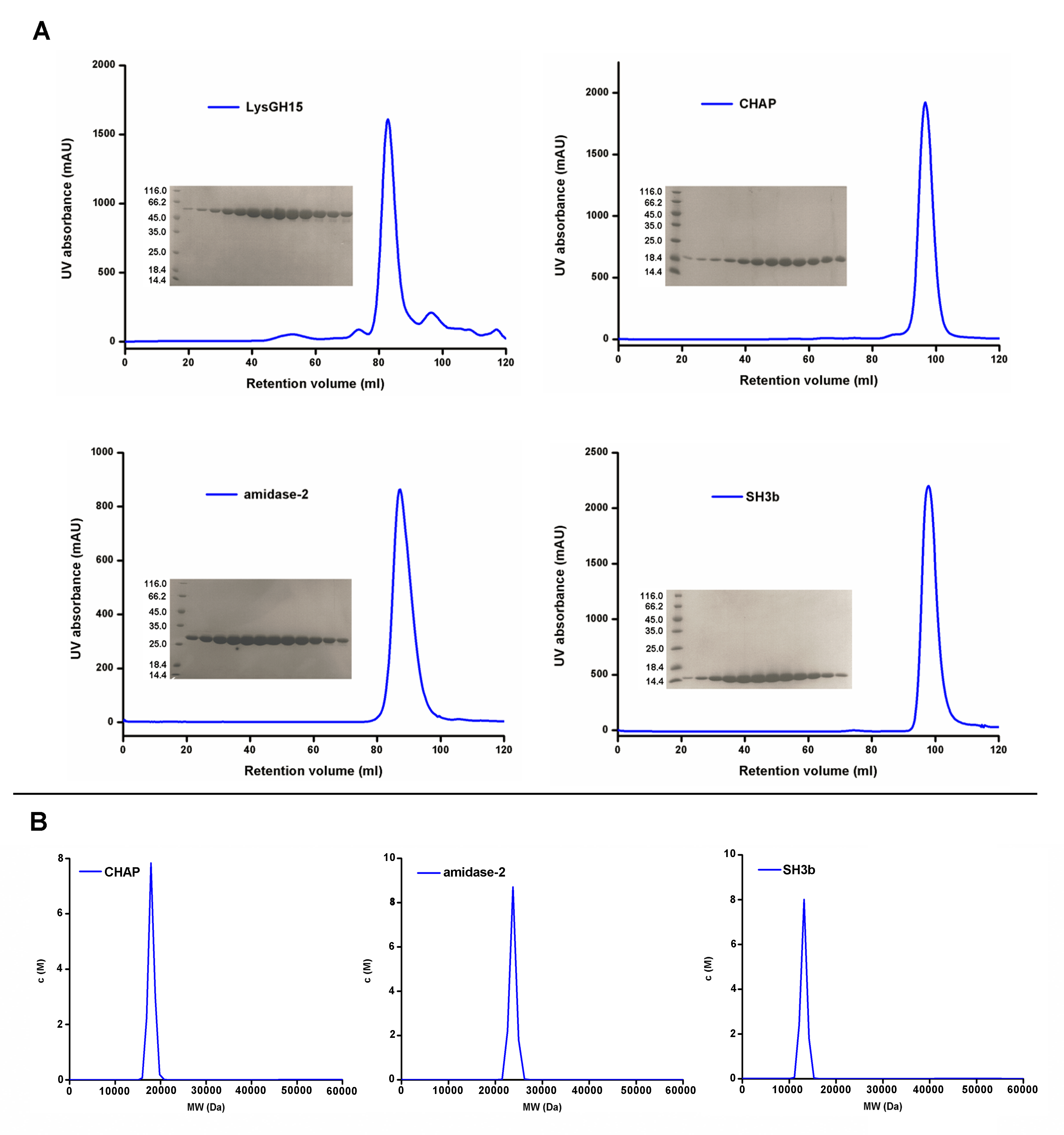

Supplement: Figure S1 — Purification of LysGH15 and its three individual domains. (A) Size-exclusion chromatography (SEC) of the full-length LysGH15 and its three individual domains (CHAP, amidase-2, and SH3b). SDS-PAGE analysis (inset) of the protein collected from each major peak. (B) Analytical ultracentrifugation (AUC) analysis of the three individual LysGH15 domains (CHAP, residues 1–165; amidase-2, residues 165–403; and SH3b, 368–495). (TIF) [file ppat.1004109.s001.tif]

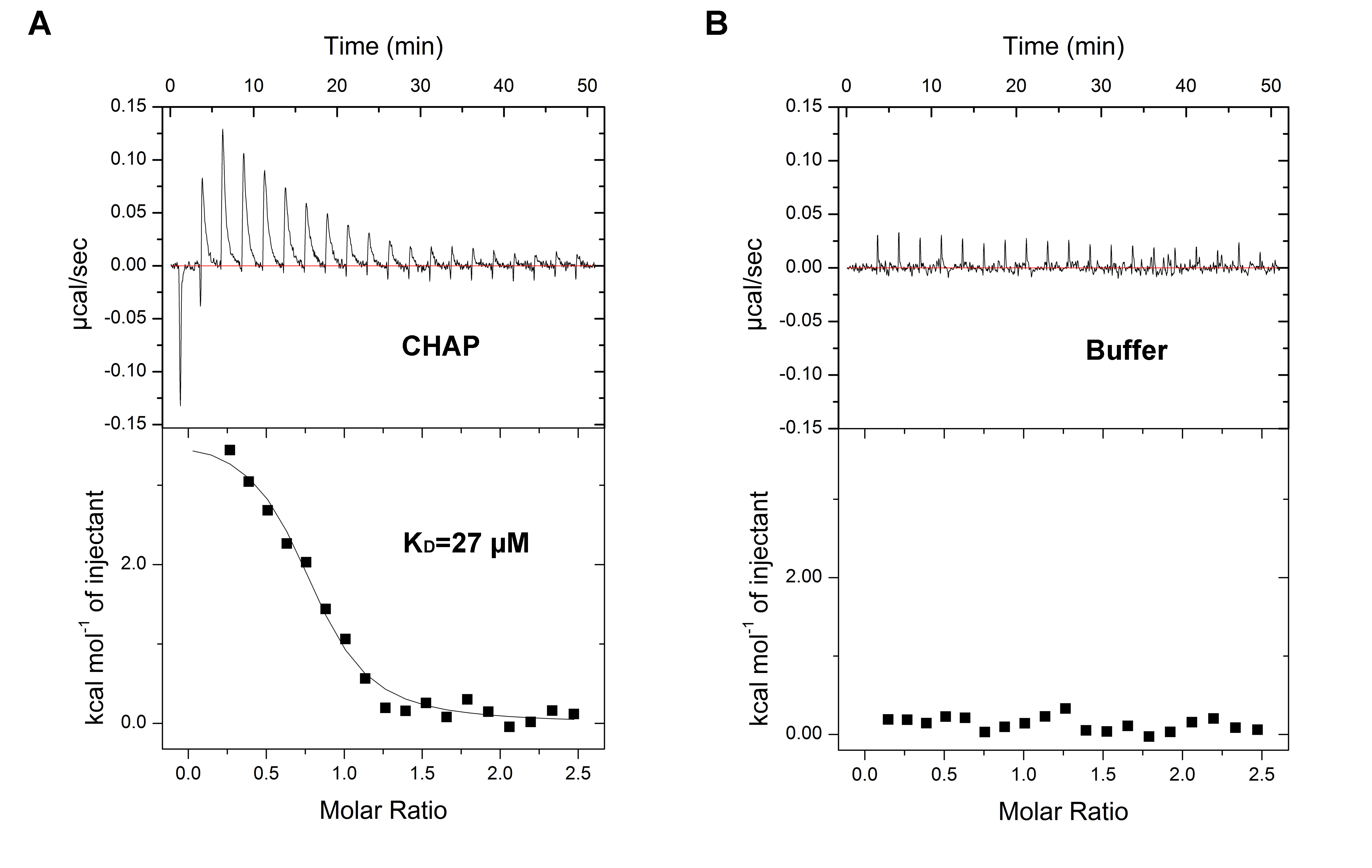

Supplement: Figure S2 — The equilibrium dissociation constant of the CHAP domain in the presence of calcium ions was determined using ITC. (A) 600 µM CaCl2 was stepwise injected into 50 µM the CHAP protein samples. (B) 600 µM CaCl2 was injected into the buffer as control. (TIF) [file ppat.1004109.s002.tif]

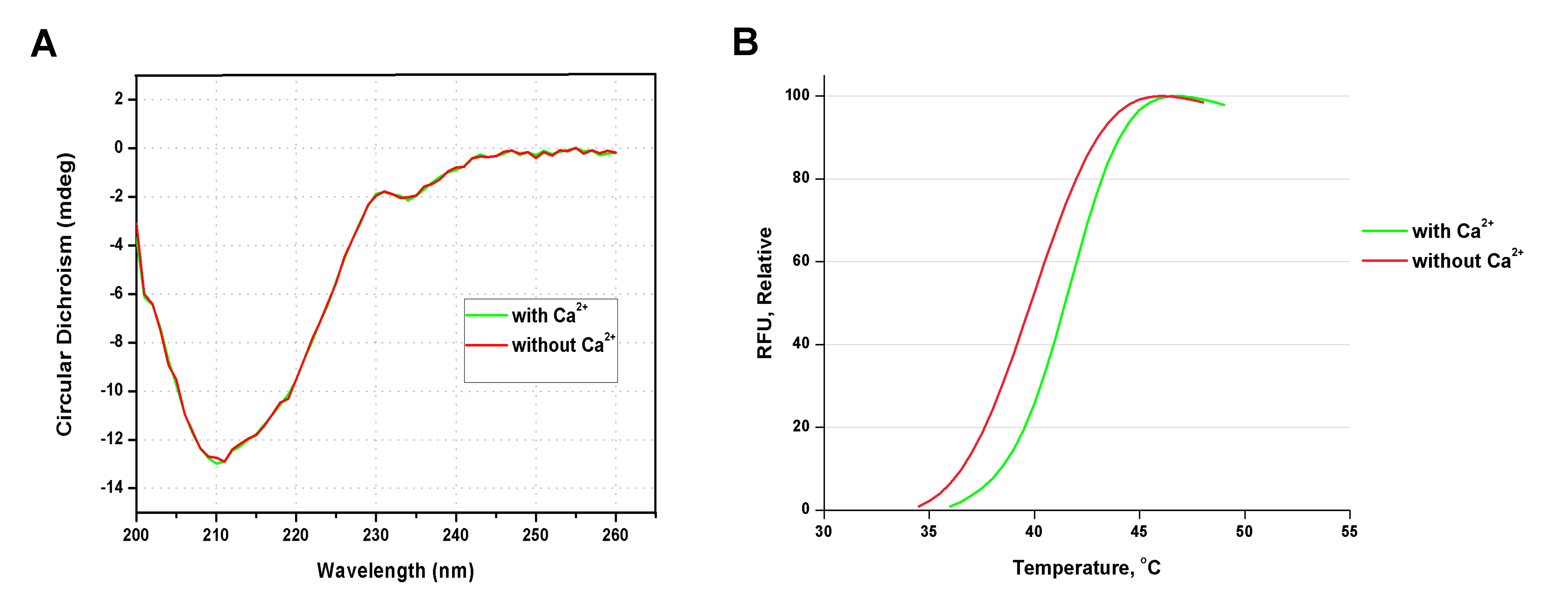

Supplement: Figure S3 — The biophysical effect of Ca2+ binding to the LysGH15 CHAP domain. (A) Circular dichroism (CD) spectroscopy of the CHAP domain with/without Ca2+. (B) The thermal shift assays of the CHAP domain with/without Ca2+. (TIF) [file ppat.1004109.s003.tif]

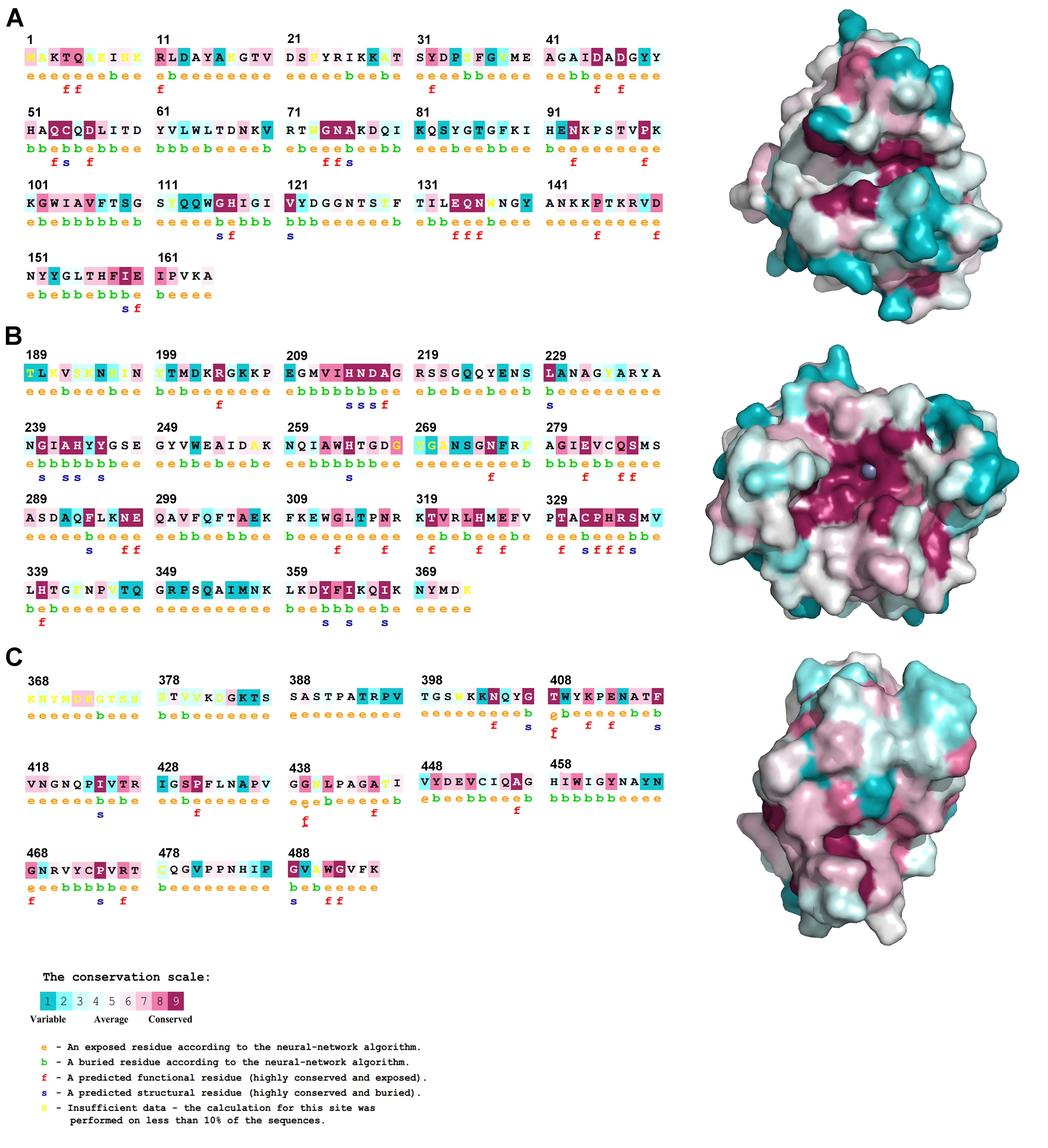

Supplement: Figure S4 — Alignment of sequences and surface rendering of the groove. (A) The CHAP domain. (Left), alignment of the 35 unique protein sequences indicating the conserved residues; (right), surface rendering of the groove in the LysGH15 CHAP domain. (B) The amidase-2 domain. (Left), alignment of the 44 unique protein sequences; (right), surface rendering of the groove in the LysGH15 amidase-2 domain. (C) The SH3b domain. (Left), alignment of the 26 unique protein sequences; (right), surface rendering of the groove in the LysGH15 SH3b domain. This figure was generated using the ConSurf server (http://consurftest.tau.ac.il/) and PyMOL [67]. (TIF) [file ppat.1004109.s004.tif]

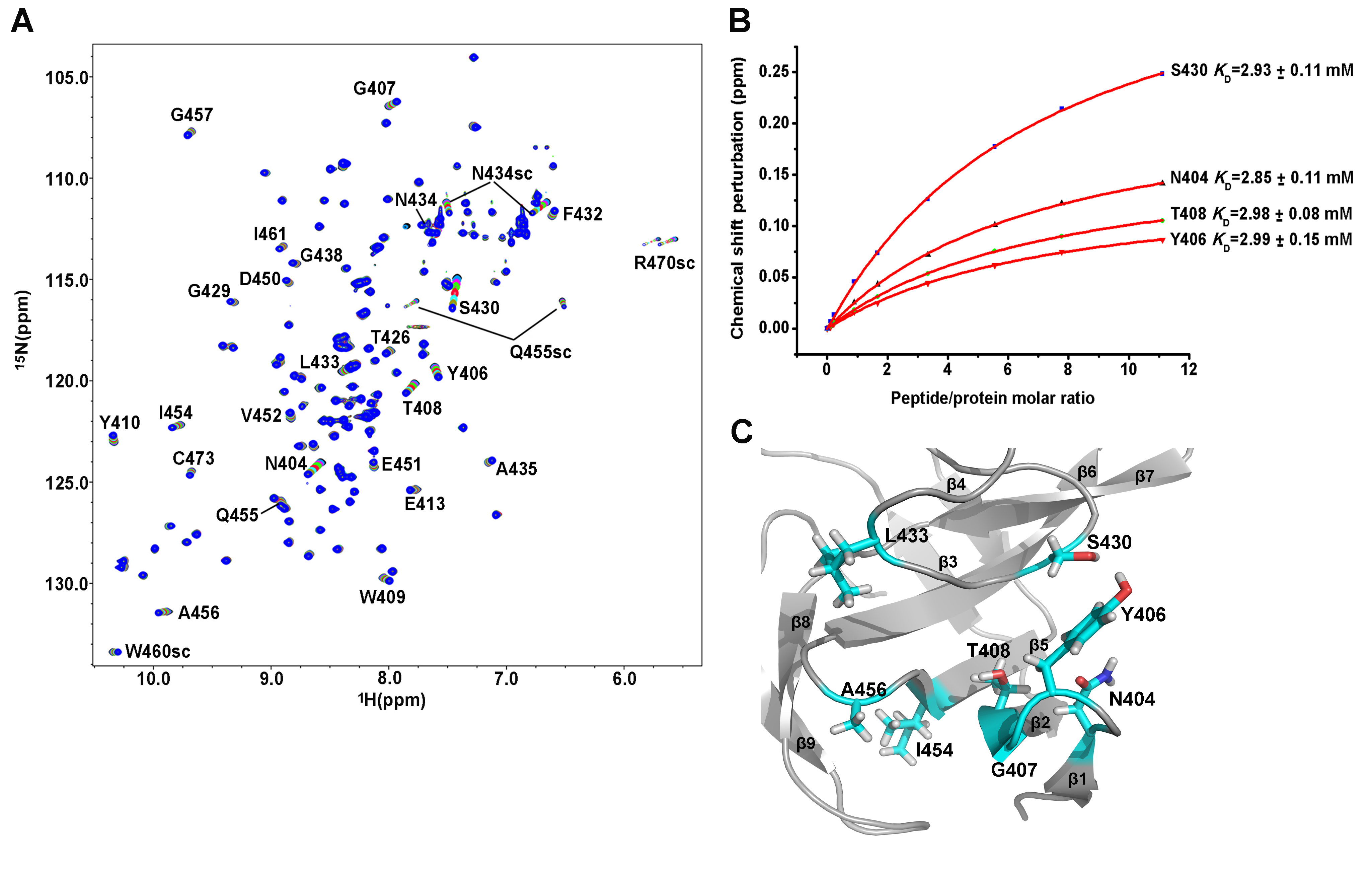

Supplement: Figure S5 — Titration of the LysGH15 SH3b domain with the peptide “AGGGGG”. (A) 1H-15N HSQC spectra of the titration of the SH3b domain with the peptide “AGGGGG”. Residues exhibiting chemical shift perturbations are labeled. (B) K D values for binding of the SH3b domain to “AGGGGG”. The fit of the four residues exhibiting the largest chemical shift perturbations are shown with the determined K D values. (C) A detailed view of the residues in the SH3b domain that interact with the “AGGGGG” peptide. The residues involved in the interaction with the peptide are shown as sticks. (TIF) [file ppat.1004109.s005.tif]
