# Supplementary material for: Structural and Biochemical Characterization Reveals LysGH15 as an Unprecedented “EF-Hand-Like” Calcium-Binding Phage Lysin
Source: PLoS Pathog. 2014 May 15;10(5):e1004109. doi: 10.1371/journal.ppat.1004109 (PMC4022735; doi:10.1371/journal.ppat.1004109)
Supplement: Table S1 — Data collection and refinement statistics. (DOC) [file ppat.1004109.s006.doc]

**Table S1.** **Data collection and refinement statistics.**

| Parameter | CHAP  (Se-Met) | CHAP  (Native) | amidase-2  (Iodide) | amidase-2  (Native) |
| --- | --- | --- | --- | --- |
| PDB ID |  | 4OLK |  | 4OLS |
| X-ray source | SSRF | SSRF | APS | SSRF |
| Crystal to detector distance (mm) | 330 | 380 | 320 | 340 |
| Number of images | 360 | 310 | 180 | 570 |
| Oscillation width (º) | 0.5 | 0.5 | 1 | 0.5 |
| Wavelength (Å) | 0.98 | 0.98 | 0.97 | 0.98 |
| Space group | *P*6_2_22 | *P*6_2_22 | *P*6_3_ | *P*6_3_ |
| a, c (Å) | 113.40, 177.92 | 113.32, 178.50 | 135.93, 106.79 | 135.40, 107.07 |
| Mosaicity (º) | 0.47 | 0.45 | 0.46 | 0.62 |
| No. of protein molecules/ASU | 2 | 2 | 4 | 4 |
| No. of phasing sites (selenium) /ASU | 4 |  |  |  |
| No. of phasing sites (iodide) /ASU |  |  | 4 |  |
| Resolution range (Å) | 50.00-2.80 | 50.00-2.80 | 50.00-2.20 | 50.00-2.10 |
|  | (2.90-2.80) | (2.90-2.80) | (2.28-2.10) | (2.18-2.10) |
| Rsym (%) cif | 13.4 (49.9) | 9.3 (40.4) | 13.7 (46.1) | 7.7 (37.4) |
| Mean I/σ(I) cif | 44 (11) | 37.70 (11.91) | 21.37 (4.09) | 41.07 (8.7) |
| Completeness (%) cif | 100 (100) | 99.7 (100) | 99.0 (91.5) | 96.9 (77.4) |
| Redundancy | 11.0 (10.9) | 18 (18.6) | 9.1 (4.6) | 16.1 (11.1) |
| **Refinement** |  |  |  |  |
| Resolution (Å) |  | 47.85-2.69 |  | 39.57-2.27 |
| No. of reflections |  | 18004 |  | 48572 |
| *R*_work_/*R*_free_ (%) |  | 17.37/20.39 |  | 15.76/16.08 |
| No. of atoms |  | 2804 |  | 6563 |
| No. of protein atoms |  | 2627 |  | 5904 |
| No. of ligand atoms |  | 21 |  | 19 |
| No. of waters |  | 156 |  | 640 |
| Wilson B (Å^2^) |  | 41.45 |  | 26.50 |
| Mean B (Å^2^) |  | — |  | 37.05 |
| **R.M.S. deviations** |  |  |  |  |
| Bond lengths (Å) |  | 0.011 |  | 0.009 |
| Bond angles (º) |  | 0.838 |  | 1.13 |
| **Ramachandran analysis** |  |  |  |  |
| Favored region (%) |  | 96.92 |  | 97.00 |
| Allowed region (%) |  | 3.08 |  | 3.00 |
| Outliers (%) |  | 0.00 |  | 0.00 |

The numbers in parentheses represent values for the highest resolution shell.
