# Supplementary material for: Structural and Biochemical Characterization Reveals LysGH15 as an Unprecedented “EF-Hand-Like” Calcium-Binding Phage Lysin
Source: PLoS Pathog. 2014 May 15;10(5):e1004109. doi: 10.1371/journal.ppat.1004109 (PMC4022735; doi:10.1371/journal.ppat.1004109)
Supplement: Table S2 — The experimental restraints and structural statistics for the 20 lowest energy structures of the LysGH15 SH3b domain. (DOC) [file ppat.1004109.s007.doc]

**Table S2. The experimental restraints and structural statistics for the 20 lowest energy structures of the LysGH15 SH3b domain.**

| PDB ID | 2MK5 |
| --- | --- |
| BMRB ID | 19752 |
| Distance restraints |  |
| Intraresidue | 1257 |
| Sequential | 807 |
| Medium | 284 |
| Long-range | 1096 |
| Ambiguous | 1297 |
| Total | 4741 |
| Hydrogen bond restraints | 82 |
| Dihedral angle restraints |  |
| φ | 84 |
| ψ | 84 |
| Total | 168 |
| Violations |  |
| Max. distance restraint violation (Å) | 0.156 |
| Max. dihedral angle restraint violation (°) | 4.19 |
| RMSD from the experimental restraints |  |
| Distance restraints (Å) | 0.0026 ± 0.0005 |
| Dihedral angle restraints (°) | 0.50 ± 0.07 |
| RMSD from the ideal geometry |  |
| Bond lengths (Å) | 0.0106 ± 0.0002 |
| Bond angles (°) | 1.10 ± 0.03 |
| Impropers (°) | 1.53 ± 0.08 |
| RMSD from the mean structure (Å) |  |
| Backbone heavy atoms |  |
| All residues* | 0.50 ± 0.05 |
| Regular secondary structure** | 0.37 ± 0.04 |
| All heavy atoms |  |
| All residues | 0.84 ± 0.05 |
| Regular secondary structure | 0.71 ± 0.06 |
| PROCHECK statistics (%) |  |
| Most favored regions | 92.8 |
| Additional allowed regions | 5.5 |
| Generously allowed regions | 0.2 |
| Disallowed regions | 1.5 |

*Residues 400-495 are included in the analysis.

**Regular secondary structure regions include residues 402-403, 409-418, 424-428, 439-441, 446-455, 457-464, 470-480, 485-490, and 493-494.
